# Experimental setting and protocol impact human colour preference assessment under multiple white light sources

**DOI:** 10.3389/fnins.2022.1029764

**Published:** 2022-10-28

**Authors:** Xue Deng, Yixuan Liu, Baolin Tian, Wei Zhang, Feng Yu, Qiang Liu

**Affiliations:** ^1^Research Center of Graphic Communication, Printing and Packaging, Wuhan University, Wuhan, China; ^2^Key Laboratory of Blockchain on Agricultural Vegetables, Weifang University of Science and Technology, Weifang, China; ^3^Department of Psychology, College of Philosophy, Wuhan University, Wuhan, China

**Keywords:** psychophysics, colour preference, experimental protocol, sex difference, light source

## Abstract

Psychophysical experiment is the most straightforward and reliable way to investigate the impact of lighting on visual colour perception. In this study, a series of experiments were conducted in order to investigate the impact of experimental setting and protocol on the obtained conclusions in visual tests regarding human preference on object colour in applied lighting research. Four light sources of 5,500 K, with Duv values of −0.01, 0, 0.015, and 0.02, were used to illuminate different kinds of objects including blue jeans, fruit and vegetables, bread, artware, fresh pork, and skin tones. The use of those experimental light sources and objects was to provide control study for our former research by deliberately changing certain experimental setup and protocol and testify the robustness of our former conclusions. The results show that some of our former findings, like the dominant impact of lighting on colour preference, the visual cognition process of light booth experiments as well as the correlation between the whiteness of lighting and colour preference, were found to be valid in typical light booth experiment. The impact of experimental object turned out to be much stronger under the newly designed protocol and the significance of sex difference on colour preference judgment was found to vary with experimental setup. These new findings highlight the influence of experimental setting and protocol on the validity of research findings, which we believe, could provide deeper understanding for the psychophysical results of current colour preference studies.

## Introduction

With the rapid development of LED technology, colour quality of lighting has become a hot topic among lighting industry. Researchers have investigated the colour rendition performance of white light sources from many visual aspects including colour fidelity (Nickerson and Jerome, 1965; Davis and Ohno, 2010), colour preference (Huang et al., 2017, 2019a,2019b; Yixuan Liu et al., 2017a), colour naturalness (Jost-Boissard et al., 2009, 2015), colour vividness (Khanh and Bodrogi, 2016; Khanh et al., 2016, 2017), colour harmony (Szabó et al., 2009), and colour discrimination (Thornton, 1972; Jiang et al., 2015). Among those visual attributes, colour preference of lighting (Smet et al., 2011; Khanh and Bodrogi, 2016; Khanh et al., 2016, 2017; Huang et al., 2017; Liu et al., 2017a), which refers to the visual appreciation of human observers on the colour appearance of illuminated objects, is perhaps the most conspicuous issue among the above-mentioned visual attributes. Such an issue is closely associated with the second top priority topic “Colour Quality of Light Sources Related to Perception and Preference” in the Commission Internationale de l’Eclairage (CIE) Research Strategy (CIE, 2020). Meanwhile, CIE has also established a Research Forum, RF-03 (Matters Related To Colour Rendition) and encouraged worldwide researchers to further investigate this topic.

Till now, numerous studies have been carried out to investigate colour preference under different lighting, in which subjects were invited to rate their visual preference on the colour appearance of illuminated objects under different white light sources. However, consensus has not been reached upon which object should be adopted to evaluate the colour rendition performance. Currently, various objects have been used including fruit and vegetables (Jost-Boissard et al., 2009, 2015), skin tones (Islam et al., 2013; Wei et al., 2014; Tan and Stephen, 2019), artwork (Nascimento and Masuda, 2014; Zhai et al., 2014; Huang et al., 2017, 2020a), printed images (Islam et al., 2013), cosmetics (Khanh and Bodrogi, 2016), consumer goods (Khanh et al., 2016) as well as combined objects (Royer et al., 2016; Khanh et al., 2017). Many researchers preferred to ask the subjects to observe familiar objects like fruit, vegetables, and bread (Thornton, 1974; Jost-Boissard et al., 2009; Wang and Wei, 2018) but others used unfamiliar objects like artwork (Nascimento and Masuda, 2014) and printed images (Islam et al., 2013).

In our previous work (Huang et al., 2017), we systematically evaluated human colour preference upon 14 groups of objects including four groups of fruit and vegetables with different colours, five traditional Chinese calligraphies written on papers with different colours, four pieces of artwork as well as a bouquet of artificial flowers with mixed colours. Those objects were all illuminated by the same group of white light sources with Correlated Colour Temperature (CCT) values ranged from 2,500 to 6,500 K, under an illuminance level of 200 lx. The experimental results showed that the rating intervals of the observers for the familiar objects were obviously larger than that of unfamiliar objects, which revealed the impact of observer familiarity upon colour preference evaluation under different lighting.

Meanwhile, the most significant finding of the above study is the dominant impact of lighting on colour preference evaluation. That is, no matter what kind of objects were posed in the viewing booth, the colour preference ratings of observers under different light sources exhibited a very similar trend (Huang et al., 2017). That finding was further consolidated by a follow-up study (Liu et al., 2017a) in which we examined the same group of light sources with the same experimental setting and protocol but put no objects in the booth. Interestingly, the average preference ratings of observers for the lit environment of the empty booth under multiple light sources were highly consistent with the colour preference scores for multiple illuminated objects, as reported before (Huang et al., 2017). Such consistency inspired us to reconsider the impact of experimental setting and protocol on the final conclusions obtained in similar psychophysical tests in booth.

Thus, based on a meta-analysis of eight groups of visual data from worldwide researchers, recently we have demonstrated that the perceived colour preference obtained by consecutive visual judgments in typical light-booth experiments was greatly influenced by the inherent features of the visual cognition process rooted in that kind of experiments (Chen et al., 2020a). Specifically, during the psychophysical tests, after several rounds of visual judegments, observers gradually realised that they were observing the same objects and the research variable was lighting. This knowledge would help their visual system to further discount the illuminant and produce a relatively consistent colour perception for the objects. Thus, for the remaining part of the test, it is likely that the observers paid more attention, consciously or unconsciously, to the lit environment of the booth rather than the objects. Such a finding could well explain the dominant influence of lighting on colour preference (Huang et al., 2017) as well as the consistency between colour preference ratings under different light sources with and without experimental objects in booth (Liu et al., 2017a). Obviously, that research further demonstrates the non-negligible impact of experimental setting and protocol on the final psychophysical results.

Based on the above considerations, we revisited our former work that evaluated multiple experimental objects (Huang et al., 2017) and realised that the preponderant impact of lighting over experimental objects might be attributed to the specific experimental setting and protocol: in that study the experimental light sources were remarkably different and they were directly compared in every experiment while different kinds of objects were not compared in the same trial (i.e., they were separately assessed in different sub-tests). This leads to the fact that visual scores obtained in different sub-group studies with different kinds of experimental objects were independent with each other, which means that the influence of objects was to some extent weakened by that setting and protocol.

Another conspicuous finding we obtained after revisiting our former psychophysical data (Huang et al., 2017, 2018, 2019a, 2020a, 2020b, 2021b; Chen et al., 2020b; Liu et al., 2020; Wang et al., 2020) is about sex difference. In our former studies, various objects were adopted in light booth experiments, including blue jeans (Huang et al., 2018; Liu et al., 2020), paintings (Huang et al., 2017, 2020a), artware (Wang et al., 2020), calligraphy (Huang et al., 2017), black and white objects (Huang et al., 2019b), fruit and vegetables (Huang et al., 2017, 2021b), bread and cakes (Chen et al., 2022), and so on, and they were illuminated by different groups of white light sources. In every visual study, equal amount of male and female observers were invited. In this study, we used a Mann–Whitney *U* test to investigate whether the colour preference between men and women for certain object under certain light source was significantly different (*p* < 0.05). Surprisingly, for all the 357 lighting scenarios (visual tests with a certain combination of test object and test light source) depicted in our former papers ([Huang et al., 2017, 2018, 2019a, 2020a, 2020b, 2021b; Chen et al., 2020b; Liu et al., 2020; Wang et al., 2020), significant sex difference was only observed in the work that related to blue jeans (Liu et al., 2020). In that study, males and females judged the colour appearance of seven pairs of jeans consecutively and their responses were significantly different for all jeans under two specific lighting conditions: male observers provided significantly higher scores than females under two light sources of 5,500 K, with Duv values (the distance from the test chromaticity coordinates to the Planckian locus) of 0.02 and 0.015, respectively. Interestingly, in that study such sex difference was not found under the remaining 5,500 K light sources with other Duv values.

Based on the above findings, in this study we deliberately designed a control experiment to discuss the potential impact of experimental setting and protocol on colour preference evaluation and testify the external validity of our former conclusions. Four light sources of 5,500 K used in our jeans study (Liu et al., 2020) were adopted and their Duv values were 0.02, 0.015, 0 and −0.01, respectively. Among those light sources, sources with Duvs of 0.02 and 0.015 refer to the cases where significant sex difference was observed, the one with a Duv of −0.01 was reported to obtain highest average colour preference scores in the former research (Liu et al., 2020) and the light source with a Duv of 0 represents the most typical 5,500 K light that locates on the blackbody locus. Meanwhile, six kinds of objects, including the formerly-used blue jeans (Huang et al., 2018; Liu et al., 2020), fruit and vegetables, bread, artware, fresh pork, and skin tones were used in the new experiment and those objects were consecutively observed in a same visual test. As a control of our previously studies (Huang et al., 2017, 2019a,2019b,2021b; Chen et al., 2020a; Liu et al., 2020), in this work we wanted to investigate:

1) Whether the formerly-reported dominant influence of lighting on colour preference (Huang et al., 2017) still exist when multiple experimental objects were assessed consecutively in one test? Similarly, we also want to find out whether the impact of experimental objects on colour preference grow stronger when they were compared directly in the same visual test?

2) Whether the light sources that generated significant sex difference for jeans perception (Liu et al., 2020) could lead to significant sex difference for other objects as well?

3) Whether our former conclusions stand when the experimental setting and protocol change? For instance, the impact of object familiarity on colour preference evaluation (Huang et al., 2017), validity of the theoretical explanation on the visual cognition process in typical light booth experiment (Chen et al., 2020a), correlation between the whiteness of lighting and colour preference (Huang et al., 2019a,b) as well as the human preference on light sources with negative Duv values (Huang et al., 2021b).

## Materials and methods

### Participants

The experiments were approved by the ethics committee of Wuhan University. All subjects provided written informed consent in accordance with the Declaration of Helsinki.

Forty observers, 20 males and 20 females, were invited to participate in the experiment, all of whom were students from Wuhan University. The age of the observer was between 19 and 34 years old, with a mean age of 22 and a standard deviation of 3.49. All of them had passed the Ishihara Colour Vision Test and they had no knowledge about the research intent before the visual test.

### Light sources

**FIGURE 1:**
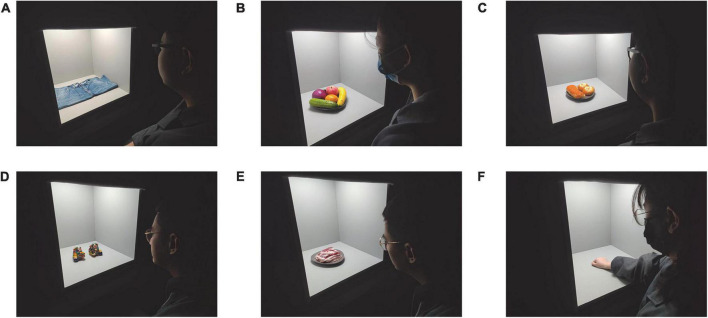
Multiple objects adopted in the psychophysical experiments. **(A)** Blue jeans, **(B)** Fruit and vegetables, **(C)** Bread, **(D)** Artware, **(E)** Fresh pork, **(F)** Skin tones.

The visual experiment of this study was conducted in a light booth. The size of the booth is 50 cm × 50 cm × 60 cm (W × D × H). The walls and bottom of the booth were evenly coated with Munsell N7 neutral gray matte paint. A height-adjustable chair was used to ensure that the illumination source at the top of the light booth was not visible to the observers. The chair was placed at a distance of approximately 50 cm from the light booth, which resulted in a viewing angle of approximately 45°. The experimental scenes are shown in Figure 1.

**FIGURE 2:**
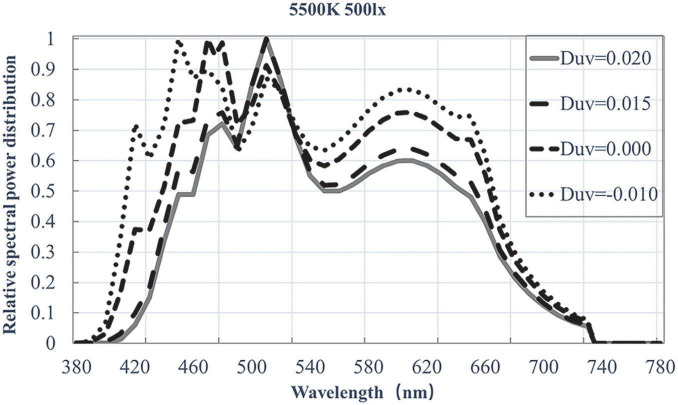
Relative spectral power distributions of experimental light sources.

The experimental light sources were generated by a spectrally tunable lighting system developed by Changzhou Thouslite Ltd., Changzhou, China. As mentioned earlier, the four light sources were of 5,500 K and their Duv values were −0.01, 0, 0.015, and 0.02, respectively. The illuminance level at the bottom of the booth was uniformly set to 500 lux, calibrated by a Testo 540 illuminance meter. The spectral power distributions (SPDs) of the light sources were measured by an X-Rite i1 Pro2 spectrophotometer, as shown in Figure 2. The colorimetric parameters of those light sources, together with their values of typical colour quality measures, are depicted in Table 1.

**TABLE 1:** The colorimetric properties of the spectral power distributions (SPDs) and their typical colour quality metric values.

CCT (K)	5,509	5,514	5,508	5,517
Duv	0.0205	0.0157	0.0007	−0.0093
X	0.332	0.332	0.332	0.332
Y	0.384	0.373	0.342	0.323
u’	0.191	0.195	0.206	0.213
v’	0.497	0.492	0.478	0.468
CRI (Ra)	89	90	89	91
GAI	75	80	94	105
Rf	83	85	89	95
Rg	92	94	98	104
MCPI	91	96	108	118

(x, y), CIE 1931 chromaticities; (u’, v’), chromaticities in CIE 1976 colour space; Duv, distance from the testing chromaticity to the blackbody locus; CRI-Ra/R9, the CIE general/special colour rendering index (Nickerson and Jerome, 1965); GAI, gamut area index (Freyssinier and Rea, 2010); Rf (colour fidelity score) and Rg (colour gamut score), IESNA TM-30 metrics (David et al., 2015); MCPI, colour preference index based on meta-analysis (Huang et al., 2021a).

### Experimental objects

The six types of experimental objects, including blue jeans, fruit and vegetables, bread, artware, fresh pork, and skin tones, were deliberately selected and each of them exhibited unique colour features. The jeans adopted in this work had been used in our former research (Huang et al., 2018; Liu et al., 2020) and it represented monochromatic and typical costume colours. Fruit and vegetables and artware are of colourful and saturated colours. The former refers to familiar colours while the latter refers to unfamiliar colours. Bread, fresh pork, and the observer’s own skin colour are of different dominant tones and in this cases, most student observers are familiar with the colour of bread and skin but they are not familiar with the fresh pork colour. During the test, fruit and vegetables, bread, and fresh pork were purchased from the same supermarket every day to maintain freshness and thus keep colour consistent. Noteworthily, for skin tones, observers evaluated the colour of their own skin in the back of his/her right hand. During the test, those objects were placed in the middle of the light booth, as shown in Figure 1.

### Rating method

During the visual tests, participants were asked to respond with their colour preference using a 7-point rating scale, in which 1, 2, 3, 4, 5, 6, and 7 denotes strongly dislike, moderately dislike, slightly dislike, neutral, slightly like, moderately like, and strongly like, respectively. In addition to this, during each test a randomly selected light source was displayed twice without informing the participants. Such a setting was aimed for quantifying the intra-observer variability of each observer.

### Procedure

Upon arrival, subjects were asked to participate in a personal information survey and fill in their name, sex, age, major, and hometown on a questionnaire. Afterward, they were instructed to sign a consent form and screened privately for colour vision deficiencies using an Ishihara Colour Vision Test. The qualified participant was then asked to wear a grey coat, escorted to the laboratory and instructed to sit in front of the light booth. He/she was asked to adjust the height of the chair so that the light source at the top of the booth cannot be seen directly. The experimenter then introduced the visual experiment and answered questions raised by the participants. These steps were proceeded orally, with the aim of avoiding the influence of reflected light on the observer’s visual adaptation condition, which might be caused if the observer was to write the answers onto a piece of white paper. Afterward, the experimenter turned off the lights of the room in the laboratory, leaving the light source in the booth the only illumination in the room.

Before the formal experiment, the participant was provided with 30 s to visually adapt to a welcome light, which was randomly selected from the 4 experimental light sources. Afterward, a training session was provided in which the observer was asked to rate his/her preference on the colour appearance of a randomly selected object placed in booth. This step was to help the observer become familiar with the 7-point rating method.

After the training process, the formal experiment began. The participant was required to close the eyes for 20 s when the experimenter turned on the first light source. Subsequently, the observer was instructed to open the eyes and observe the lit environment of the empty booth for 30 s for visual adaption. After that, the experimenter posed the first object in the booth and asked the participant to give his/her preference score according to the colour appearance of the illuminated object. During this step, the observer was provided with sufficient time (no less than 15 s) to make the judgement.

When the participant responded with his/her assessment, the experimenter asked him/her to keep the eyes closed for another 20 s. During that time, the experimenter recorded the visual score and switched to the next light. Note that every time the light sources changed, the observers were instructed to close the eyes for 20 s, such a step was designed to eliminate the short-term memory effect of the previous lighting. The above procedure was repeated until the participant completed evaluation for the first object under 5 (4 experimental lights + 1 repeated trial) light sources. Then, the participant was asked to close the eyes and the experimenter changed the object. This procedure was repeated for each of the lights and then for each object, which means that participants had to rate with their colour preference of one object under different lights and then repeat the trial for a second object. Note that during the test, the presenting order of objects and light sources was randomised and counterbalanced among observers. In the experiment, the position of each kind of objects in the booth was fixed. On average, each participant took approximately 50–70 min to complete the experiment.

### Data analysis methods

#### Standardised residual sum of squares values

As stated above, to quantify the intra-observer variability, participants were required to rate a randomly selected light source twice without being informed of this. Thus, for each object, the standardised residual sum of squares (STRESS) values (Melgosa et al., 2011) between the two ratings were then calculated for quantifying the intra-observer variability. To assess the inter-observer variability, for each object the STRESS values were calculated between each observer’s ratings and those of the average observers.

#### Wilcoxon signed-rank test

In order to explore whether colour preference scores has significant differences between positive Duvs (Duv = 0.020, Duv = 0.015) and non-positive Duvs (Duv = −0.010, Duv = 0), Wilcoxon signed-rank test was performed on the visual data of all the experimental objects. To discuss whether the rank order of human preference for different objects changes with the experimental light source, the statistical difference between the preference scores for different objects are also investigated by such a test.

#### Pearson correlation coefficients

Pearson correlation coefficients were computed in order to assess the relationship between the mean of each object and its corresponding standard deviation. Meanwhile, it was also used to further demonstrate whether there was a significant correlation between whiteness ratings and colour preference ratings.

#### Repeated measures analysis of variance

A repeated measures analysis of variance (rm-ANOVA) was conducted in order to investigate the effect of different contextual factors including Duv (light sources), objects, sex and their interaction on colour preference ratings.

## Results

### Observer variability

Table 2 summaries the average values and standard deviations of intra- and inter-observer variability for each object. As can be seen from the table, The STRESS values for intra-observer variability ranged between 21.43 and 28.52 for the six scenarios with different objects, with a mean of 25.49. For inter-observer variability, the STRESS values ranged between 13.36 and 20.05 for the six scenarios, with a mean of 17.19.

**TABLE 2:** Inter- and intra-observer variability.

Object	STRESS	Mean	Standard deviation
Blue jeans	Inter-observer	18.60	9.72
	Intra-observer	28.39	–
Fruit and vegetables	Inter-observer	13.36	6.93
	Intra-observer	21.43	–
Bread	Inter-observer	16.04	8.09
	Intra-observer	27.35	–
Fresh pork	Inter-observer	20.05	10.78
	Intra-observer	28.52	–
Artware	Inter-observer	17.36	9.17
	Intra-observer	23.12	–
Skin tones	Inter-observer	17.72	10.64
	Intra-observer	24.14	–
Overall	Inter-observer	17.19	–
	Intra-observer	25.49	–

The mean value and standard deviation were computed based on the observer variabilities of the four experimental light sources.

Generally speaking, a STRESS value no larger than 30 is usually considered as reasonable for visual experiments (Melgosa et al., 2011). Therefore, as depicted in Table 2, the intra- and inter- observer variability of this study can be regarded as acceptable. For inter-observer variability, the results are consistent with our former research (Huang et al., 2021b) (STRESS = 24.2, standard deviation = 8.5) and the work of Feltrin et al. (2019) (STRESS = 25.7, standard deviation = 7.9). It seemed that aesthetic attributes and familiarity mutually impacted observer variability: for fruit and vegetables with higher degree of aesthetic attributes and familiarity, the observer variabilities were low, while for fresh pork with lower degree of aesthetic attributes and familiarity, the observer variabilities were high. As for the other four objects, they only possessed one of these two attributes thus the observer variability was medium.

### Overall results

**FIGURE 3:**
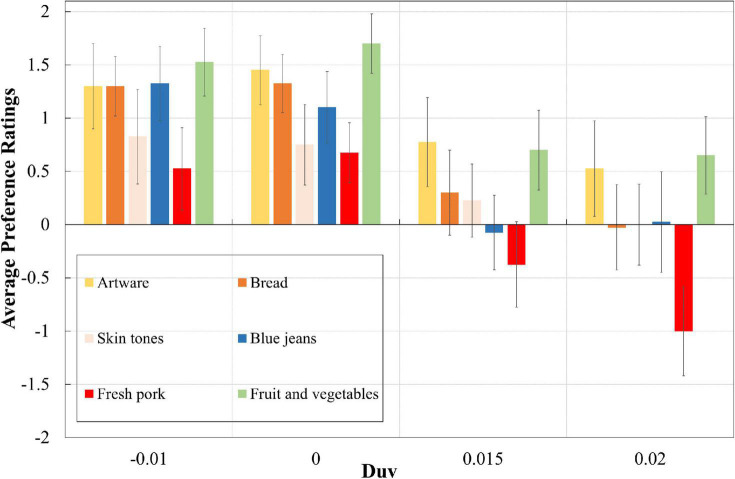
Preference scores of six experimental objects under four light sources. The error bars denote the 95% confidence intervals.

Figure 3 summarises the average preference ratings of six types of experimental objects under four light sources with different Duv values. From this drawing, it is clear that no matter under which light sources, observers preferred the colour appearance of artware and fruit and vegetables while they did not appreciate the appearance of fresh pork. Similarly, despite of experimental objects, light sources with zero and negative Duv values always received higher scores than sources with positive Duvs. According to a Wilcoxon signed-rank test, for almost every experimental object, there was a significant difference (*p* < 0.05) in colour preference scores between positive Duvs (Duv = 0.020, Duv = 0.015) and non-positive Duvs (Duv = −0.010, Duv = 0). The only exception is the colour appearance of artware under sources with Duv values of −0.01 and 0.015, but the difference between those colour preference scores was also obvious, with a significance level of *p* = 0.07. Those results indicated that in this situation the impact of object and light sources on colour preference was strong and independent, with no interactive effect. Meanwhile, it was found that for every object, the average scores and standard deviations of the preference ratings were always strongly and negatively correlated, with Pearson correlation coefficients closed to −1, which is consistent with our formerly-reported results (Huang et al., 2017).

As can be seen from Table 3, repeated measures ANOVA for the colour preference ratings showed significant main effects for Duv (light sources) [*F*_(3, 114)_ = 49.435, *p* < 0.001, η^2^ = 0.565] and objects [*F*_(5, 190)_ = 12.715, *p* < 0.001, η^2^ = 0.251]. The partial eta-squared values (η^2^) reveal the magnitude of influence and it is clear that the impact of lighting and object is much stronger than other factors, since the η^2^ values are much larger than those of other factors. And just like our former conclusions (Huang et al., 2017), light exhibited dominant influence.

**TABLE 3:** Statistical significance of the effect of the independent variables (Duv, object, and sex) on the dependent variable (preference ratings).

Independent variable or interaction	SS	df	MS	F	Sig.	η^2^
Duv	249.445	1.824	136.783	49.435	**<0.001**	**0.565**
Object	144.834	4.465	32.436	12.715	**<0.001**	**0.251**
Sex	1.134	1	1.134	0.21	0.649	0.006
Duv × Object	22.836	9.257	2.467	1.701	0.085	0.043
Duv × Sex	1.52	1.824	0.833	0.301	0.721	0.008
Object × Sex	19.284	4.465	4.319	1.693	0.147	0.043
Duv × Object × Sex	18.686	9.257	2.019	1.392	0.188	0.035

SS, sum of squares; MS, mean square. The bold values denote statistical significance.

The average colour preference scores obtained in this study were compared to the average whiteness ratings for the same experimental light sources acquired in our former psychophysical test carried out in the same booth (Huang et al., 2019b). In that study, 30 observers were invited to rate their whiteness perception (i.e., neutrality: a colour that neither green nor red, nor yellow or blue) for the lit environment in the same empty light booth. The same nine experimental light sources (5,500 K, 500 lx, Duvs ranged from −0.02 to 0.02 with 0.005 interval) used in our former jeans experiment (Liu et al., 2020) were examined and those lights included the four light sources used in this study. Meanwhile, in that study, consistent experimental setting and protocol, as well as ratings approach (i.e., 7-point rating) were adopted as well.

**FIGURE 4:**
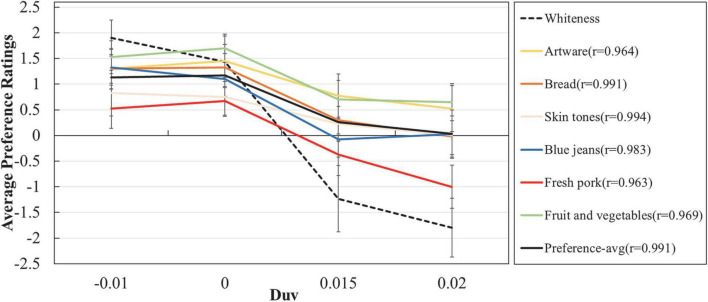
The average whiteness ratings and colour preference ratings. The error bars are the 95% confidence intervals and the values inside the brackets in the legend denote the Pearson correlation coefficients between colour preference scores and whiteness ratings.

It can be seen from Figure 4 that the trends in colour preference scores under different light sources were consistent across multiple objects, which again, highlights the dominant influence of lighting on visual appreciation (Huang et al., 2017). Most importantly, from Figure 4 we could observe that the colour preference scores of multiple objects were highly correlated with the whiteness ratings, with Pearson correlation coefficients exclusively larger than 0.96. We believe that this strong correlation should essentially be attributed to the fundamental belief in visual science that the human visual system is optimised for the environment in which it evolved (Párraga et al., 2000; Pearce et al., 2014). As reported by Smet (2018), the whiteness regions for lighting are highly related to daylight scope and maybe that is the reason why human observers preference the corresponding lit environment. As shown in the Figure 4, the rating interval of whiteness scores regarding to different lighting was larger than those of colour preference scores for different objects. Such results agree well with our former results (Huang et al., 2019b,2021b), which indicate that it is easier for human observers to raise an explicit answer for whiteness perception than colour preference. Meanwhile, light sources with positive Duv values always corresponded to larger variations for preference scores of different objects.

### Object difference

From Figures 3, 4, it is easy to conclude that although the impact of lighting on colour preference ratings is dominant, the rank order of human preference toward different experimental objects does not vary pronouncedly with light sources: artware and fruit and vegetables were most preferred while fresh pork was least preferred, no matter under which light sources. Such conclusion is further clarified by Table 4, in which the statistical difference between preference scores of different objects was demonstrated by the results of a Wilcoxon signed rank test.

**TABLE 4:** Wilcoxon signed rank test results for every two objects.

Object	Duv							
	0.020		0.015		0.000		−0.010	
	*Z*	*P*	*Z*	*P*	*Z*	*P*	*Z*	*P*
Blue jeans × Fruit and vegetables	−2.364^b^	**0.018**	−3.127^b^	**0.002**	−2.581^b^	**0.01**	−0.665^b^	0.506
Blue jeans × Bread	−0.099^b^	0.921	−1.570^b^	0.116	−0.693^b^	0.488	−0.160^b^	0.873
Blue jeans × Pork	−3.504^b^	**<0.001**	−1.148^b^	0.251	−1.978^b^	**0.048**	−2.662^b^	**0.008**
Blue jeans × Artware	−1.746^b^	**0.081**	−2.884^b^	**0.004**	−1.578^b^	0.114	−0.021^b^	0.983
Blue jeans × Skin tones	−0.011^b^	0.992	−1.082^b^	0.279	−1.589^b^	0.112	−1.560^b^	0.119
Fruit and vegetables × Bread	−3.109^b^	**0.002**	−1.920^b^	**0.055**	−1.864^b^	**0.062**	−1.081^b^	0.28
Fruit and vegetables × Pork	−4.683^b^	**<0.001**	−3.139^b^	**0.002**	−4.125^b^	**<0.001**	−3.106^b^	**0.002**
Fruit and vegetables × Artware	−0.517^b^	0.605	−0.423^b^	0.672	−1.104^b^	0.27	−0.777^b^	0.437
Fruit and vegetables × Skin tones	−2.473^b^	**0.013**	−1.712^b^	**0.087**	−3.555^b^	**<0.001**	−1.997^b^	**0.046**
Bread × Fresh pork	−3.619^b^	**<0.001**	−2.542^b^	**0.011**	−2.997^b^	**0.003**	−2.647^b^	**0.008**
Bread × Artware	−2.021^b^	**0.043**	−1.567^b^	0.117	−0.320^b^	0.749	−0.220^b^	0.826
Bread × Skin tones	−0.090^b^	0.928	−0.487^b^	0.626	−2.409^b^	**0.016**	−1.520^b^	0.128
Fresh pork × Artware	−4.103^b^	**<0.001**	−3.521^b^	**<0.001**	−3.545^b^	**<0.001**	−2.572^b^	**0.01**
Fresh pork × Skin tones	−3.590^b^	**<0.001**	−2.519^b^	**0.012**	−0.440^b^	0.66	−1.228^b^	0.219
Artware × Skin tones	−2.096^b^	**0.036**	−2.024^b^	**0.043**	−2.578^b^	**0.01**	−1.423^b^	0.155

^b^Based on positive ranks. The bold values denote statistical significance.

Meanwhile, it is interesting to find that the impact of familiarity on ratings intervals, which was reported in our former work (Huang et al., 2017), was not observed in this study. In our previous work, under light sources with lower ratings, observers rated the familiar objects with lower scores compared to unfamiliar objects while under sources with higher ratings, they provided higher scores for familiar objects than unfamiliar objects. As illustrated in Figures 3, 4, however, there is no such trend among the six experimental objects: objects that were preferred by observers received higher scores under all of the light sources.

### Sex difference

**FIGURE 5:**
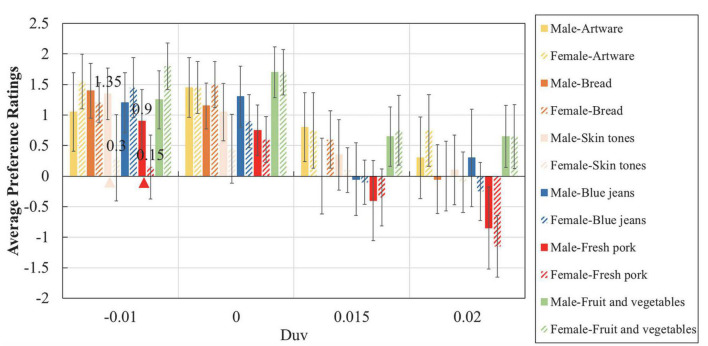
Sex difference in colour preference ratings. The error bars denote the 95% confidence intervals and the triangles indicate significant difference (*p* < 0.05) according to a Mann–Whitney *U* test (skin tones: *U* = 134.500, *p* < 0.01; fresh pork, *U* = 130.000, *p* = 0.050).

Figure 5 illustrates the sex difference in colour preference ratings obtained in this psychophysical work. As can be seen from the figure, significant sex difference was only observed when skin tones and fresh pork were illuminated by the light source with a Duv value of −0.010. As for light sources with positive Duvs, which were reported to cause significant sex difference for seven pair of jeans in our former work (Liu et al., 2020), they did not generate any significant sex difference in this case. Noteworthily, the experimental jeans adopted in this study had been used in our former work (Liu et al., 2020) and illuminated by the same light sources with Duv values of 0.015 and 0.02. In the previous condition, significant sex difference was found but this time, such difference was not pronounced.

## Discussion

The rm-ANOVA results shown in Table 3 strength our former conclusions that light dominates colour preference (Huang et al., 2017), despite of the fact that the experimental light sources, objects and protocol are all different between the two studies. Note that the colour features of the six experimental objects shown in Figure 1 were remarkably different, but as illustrated in Figure 3, 4, the preferred light sources were not obviously influenced by objects. Similar results were also reported by Jost-Boissard et al. (2009) in which the authors investigated the colour quality of lighting using a paired-comparison experiment. Based on the visual results the authors argued that: “The subjects’ judgements about lighting did not seem to depend a lot on the colour of the target. This suggests that it is possible for a given type of light to give a good rendering for all the different colours, and that separate lighting is not needed for each target colour.” Those opinions seem to belie with the common understanding that colour preference of lighting varies with experimental objects, since, by definition, colour is a synthetisation of light, object and human vision. Such a contradiction reminded us to pay closer attention to the impact of experimental setting and protocol on psychophysical findings.

In one of our former publications (Liu et al., 2017a), we ascribed the above phenomenon to the subconscious effect of human vision (Pirenne, 1948). In that work, we psychophysically validated that the preference ratings for the lit environment in the empty booth were highly consistent with the judging results when different objects were posed inside the booth and assessed (Liu et al., 2017a). The core idea of our explanation was that during the light booth experiments, the foveal response that related to the conscious vision was mainly desired. However, inevitably, during the test the subconscious vision which related to the regions surrounding the fovea with much larger viewing angles, would affect the subjective response as well. Thus, if the subconscious vision regarding the lit environment of the booth was so strong that it dominated the cognitive process of preference, putting any objects in the booth may actually turn out to be insignificant for light quality evaluation. This assumption was to some extent in line with the opinions of Khanh et al. (2017). In that work, observers were asked to evaluate the colour appearance of multiple objects against white background under different light sources and the authors concluded from their results that “The chromaticity of the white tone which is seen together with the coloured objects contributes to the colour preference assessment about the coloured objects.”

Furthermore, in our recent work, we further validated that there was strong correlation between the whiteness of lighting and colour preference, not only by psychophysical results from four groups of psychophysical experiments (Huang et al., 2019b) but also by a meta-analysis of visual data from 19 visual experiments (Huang et al., 2019a). As for this work, obviously, the consistency between the whiteness of lighting and colour preference showed in Figure 4 further consolidates the above findings. Note that unlike the previous studies, in the current work the examined experimental objects were of a much wider range of diversity and they were assessed consecutively in one experiment. Thus, the current results prove the robustness of the former conclusions.

The dominant influence of lighting on colour preference, as well as the strong correlation between the whiteness of lighting and colour preference revealed in this study could be well explained by our former opinions regarding the visual cognition process of light booth experiments (Chen et al., 2020a). That is, although it is widely believed that the neutral interior of a light booth has minimum impact on the colour appearance evaluation of experimental objects, the eight groups of psychophysical data collected in our former work (Chen et al., 2020a), together with the visual results of this study shown in Figures 3, 4, convincingly demonstrate its great impact on colour preference assessment. In other words, under the typical protocol of consecutive judgement in booth, it was unavoidable that observers gradually realised the truth that the research variable was not experimental objects but light sources. Thus, after a consecutive learning process, their visual systems would discount the illuminant, produce a relatively consistent colour perception for the objects and make the judgement based on the subconscious vision regarding the lit environment of the booth. At that time, what they were mainly perceiving was actually the colour of the lit environment in booth rather than the coloured objects. Such mechanism should be fully recognised by researchers in the field of lighting quality assessment.

The visual results shown in Figures 3, 4 also corroborate the opinion that people prefer the colour appearance of illuminated objects under light sources with negative Duv values (Dikel, 2014; Ohno and Fein, 2014; Wei and Houser, 2015; Feng et al., 2016; Ohno and Oh, 2016; Wang and Wei, 2018; Liu et al., 2020; Huang et al., 2021b). Wei and Houser (2015) ascribed such result to the fact that light sources with negative Duv values usually exhibited higher scores for colour gamut and colour fidelity. That theory, however, could not explain the consistency for the preferred light sources regarding multiple objects with diverse colour attributes, as shown in Figure 4. Meanwhile, it could not explain why human observers responded differently when they assessed achromatic objects under different lighting (Huang et al., 2019b), either. Based on the above discussion, we would like to conclude that in light booth experiment where the impact of colour appearance perception was weakened by the unique experimental setting and protocol, human preference for negative Duv values should be largely attributed to the higher degree of whiteness perception for the lit neutral environment. Note also that the above conclusions are based on a prerequisite that the experimental light sources are of acceptable CRI (Nickerson and Jerome, 1965) values. Other examples could be found where light sources with much lower CRI values were used to deliberately enlarge the influence of other factor [e.g., gamut shape (Wei et al., 2016)]. That theoretical topic is beyond the scope of this study since in this work we mainly focus on practical lighting applications with qualitied CRI values.

The above results and discussions testify the robustness of some of our former findings: as reported in section “Overall results,” when the experimental setting and protocol changed in this study, our previous findings including the theoretical explanation of visual cognitive processes in typical light booth experiment (Chen et al., 2020a), the strong correlation between the whiteness of illumination and colour preference (Huang et al., 2019a,b) and human preference for light sources with negative Duv values (Huang et al., 2021b) were found to be valid.

Meanwhile, it is found that some other findings obtained in our previous work vary with the experimental setting and protocol. First of all, the effect of experimental objects on colour preference is significantly enhanced when the experimental objects were directly compared in a single visual test: in our former work with the same light sources, jeans and experimental protocol (Liu et al., 2020), the impact of experimental objects was not significant (*p* > 0.05) according to rm-ANOVA. However, due to the large diversity of experimental objects and the consecutive rating mode, in this study the influence of object turned out to be significant (*p* < 0.001) as shown in Table 3. Similarly, in Huang et al. (2017) four groups of visual test were separately carried out using a same series of light sources but different objects to investigate colour preference of lighting. As reported in that paper, only one group of test that used objects with remarkable difference (i.e., artificial flowers vs. modern oil painting) revealed significant impact of object (*p* = 0.001). Thus, it is easy to conclude that in psychophysical studies the significance of the impact of contextual factors depends on how the experiment was designed.

Secondly, familiar objects were widely used in similar lighting quality research (Jost-Boissard et al., 2009, 2015; Royer et al., 2016; Huang et al., 2017, 2018, 2021b; Liu et al., 2020) since human observers used to judge the colour appearance of object, consciously or unconsciously, based on their colour memory (Smet et al., 2010). As stated by Jost-Boissard et al. (2015), it is easier for observers to answer subjective questions on real objects with familiar colours. Meanwhile, according to Mizokami et al. (2012), familiarity also contributes to colour adaptation. These viewpoints were to some extent supported by the observer variabilities shown in Table 2. However, as described in section “Object difference,” the impact of familiarity on preference ratings intervals reported in our former work (Huang et al., 2017) was not observed in this research. As far as we are concerned, such difference should be ascribed to the fact that in the previous work (Huang et al., 2017) the experimental objects were separately assessed in different subgroups while in this work different objects were evaluated consecutively in one test. In other words, previous observers only needed to rate the colour appearance of objects in a certain group (fruit and vegetables, calligraphies, paintings, etc.) while current participants had to assess multiple objects in one test. Thus, their ratings on one kind of object were inevitably influenced by others while for the previous observers, their ratings for different objects were independent. This difference highlights again that psychophysical results rely on experimental setting and protocol.

Thirdly, as reported in section “Sex difference,” the light sources that generated significant sex difference in jeans colour preferences in our previous experiments did not result in significant sex difference for other objects in this experiment. As known to all, sex difference is a very hot topic among human-related studies. As pointed out by de Vries and Forger. (2015) males and females usually perceive the environment differently due to the differences in sensory system. Similar findings have been extensively reported by researchers from multiple research areas, including genetics (Foote et al., 2014; Vanston and Strother, 2016), neuroscience (Palmer et al., 2013), biology (Hurlbert and Ling, 2007; Schwarzkopf et al., 2011), ophthalmology (Panorgias et al., 2010), and colour science (Bimler et al., 2010; Huang et al., 2020a). However, the inherent visual mechanism of such difference is indeed complicated by the interaction of biological, environmental, evolutional, and cultural factors. Thus, till now consensus has not been reached upon which factors shape these sex differences. As stated above, the light sources with Duv values of 0.015 and 0.02 are the only two sources that exhibited significant sex difference among our visual studies (Huang et al., 2017, 2018, 2019a, 2020a, 2020b, 2021b; Chen et al., 2020b; Liu et al., 2020; Wang et al., 2020). However, in this work with different experimental settings (e.g., the use of diverse objects with distinct colour features) and protocol (e.g., the consecutive judgement for multiple objects in one test), no sex difference was observed for such “distinctive” light sources, even for the jeans that had been evaluated under the same sources before (Liu et al., 2020). Instead, as illustrated in Figure 5, the light source with a Duv of −0.01 exhibited significant sex difference for fresh pork and skin tones. These findings imply that sex difference in colour preference is not simply associated with a certain combination of light and object. Instead, it is related to a complex psychological process during the visual test, which might be influenced by multiple, unintended effects such as boredom, fatigue, learning, experience, and adaptation.

The above discussions highlight the inseparability of the psychophysical findings and the corresponding preconditions (i.e., experimental settings and protocol). After all, due to the inherent feature of psychophysical studies, it is not possible for a single study to consider all the impacting factors simultaneously. Such limitation leads to the variation and uncertainty of such kind of research. To solve this problem, it is recommended to design the psychophysical experiment more reasonably so that to enhance the internal validity of the research and report the experimental setting and protocol detailly. Meanwhile, readers should be fully aware that conclusions obtained in a single psychophysical work should be interpreted and applied with cautious. For most cases, it might not be safe to disregard the changing precondition and directly accept the opinions. As we believe, to promote external validity and draw robustness conclusions, the meta-analysis approach (Nickerson and Jerome, 1965; Smet et al., 2011; Liu et al., 2017b; Huang et al., 2019a,b, 2021a; Chen et al., 2020a) used in this topic should be encouraged (Liu et al., 2018).

## Conclusion

In this study, a series of psychophysical experiments were carried out to testify the influence of experimental setting and protocol on the obtained findings in the research filed of colour rendition of lighting. The use of the experimental light sources, objects, as well as the corresponding experimental protocol, were based on a comprehensive review of our former visual studies in the past 5 years. Through this control study, we further consolidated some of our former findings such as the dominant impact of lighting on colour preference, the visual cognition process rooted in light booth experiments, the strong correlation between the whiteness of lighting and colour preference, as well as the visual preference toward light sources with negative Duv values. Meanwhile, we also found that some of our former statements, like the significance of object difference and gender difference, did not stand when experimental setting and protocol changed. Such results highlight the inseparability between research findings and experimental setting and protocol. By this work, the authors would like to remind readers and following researchers to treat the existing psychophysical findings with more rationality.

## Data availability statement

The raw data supporting the conclusions of this article will be made available by the authors, without undue reservation.

## Ethics statement

The studies involving human participants were reviewed and approved by the Ethics Committee of Wuhan University. The patients/participants provided their written informed consent to participate in this study. Written informed consent was obtained from the individual(s) for the publication of any potentially identifiable images or data included in this article.

## Author contributions

XD and YX-L participated in the design of the study, carried out the experiment, performed the statistical analysis, and drafted the manuscript. BL-T participated in the design of the study and carried out the experiment. QL guided the research directions and ideas and drafted the manuscript. WZ and FY proposed the concept and performed the statistical analysis. All authors contributed to the article and approved the final manuscript.

## References

[B1] Bimler D. L. Kirkland J. Jameson K. A. (2010). Quantifying variations in personal color spaces: Are there sex differences in color vision? *Color Res. Appl.* 29 128–134. 10.1002/col.10232

[B2] Chen W. Huang Z. Liu Q. Pointer M. R. (2020a). Evaluating colour preference of lighting: The light booth matters. *Opt. Exp.* 28 14874–14883. 10.1364/OE.390353 32403521

[B3] Chen W. Huang Z. Rao L. Hou Z. Liu Q. (2020b). “Research on colour visual preference of light source for black and white objects,” in *Proceedings of the advanced graphic communication, printing and packaging technology*, (Singapore: Springer Singapore), 43–50.

[B4] Chen W. Wu X. Liu Z. Liu Y. Liu Q. Pointer M. R. (2022). The impact of illuminance level, correlated colour temperature and viewing background on the purchase intention for bread and cakes. *Food Qual. Prefer.* 98:104537. 10.1016/j.foodqual.2022.104537

[B5] CIE (2020). *International commission on illumination.* Available online at: http://cie.co.at/research-strategy (accessed August 27, 2022).

[B6] David A. Fini P. T. Houser K. W. Ohno Y. Royer M. P. Smet K. A. G. (2015). Development of the IES method for evaluating the color rendition of light sources. *Opt. Exp.* 23:15888. 10.1364/oe.23.015888 26193567

[B7] Davis W. Ohno Y. (2010). Color quality scale. *Opt. Eng.* 49:033602.

[B8] de Vries GJ Forger N. G. (2015). Sex differences in the brain: A whole body perspective. *Biol. Sex Differ.* 6:15. 10.1186/s13293-015-0032-z 26279833 PMC4536872

[B9] Dikel E. E. Burns G. J. Veitch J. A. Mancini S. Newsham G. R. (2014). Preferred chromaticity of color-tunable LED lighting. *LEUKOS* 10, 101–115. 10.1080/15502724.2013.855614

[B10] Feltrin F. Leccese F. Hanselaer P. Smet K. A. G. (2019). Impact of illumination correlated color temperature, background lightness, and painting color content on color appearance and appreciation of paintings. *Leukos* 16 25–44.

[B11] Feng X. Xu W. Han Q. Zhang S. (2016). LED light with enhanced color saturation and improved white light perception. *Opt. Exp.* 24 573–585. 10.1364/OE.24.000573 26832288

[B12] Foote K. G. Neitz M. Neitz J. (2014). Comparison of the Richmond HRR 4th edition and Farnsworth–Munsell 100 Hue Test for quantitative assessment of tritan color deficiencies. *J. Opt. Soc. Am. A* 31 186–188. 10.1364/josaa.31.00a186 24695168 PMC4282932

[B13] Freyssinier J. P. Rea M. (2010). “A two-metric proposal to specify the color-rendering properties of light sources for retail lighting,” in *Proceedings of the tenth international conference on solid state lighting*, San Diego, CA, 7784. 10.1117/12.863063

[B14] Huang Z. Liu Q. Liu Y. Pointer M. Luo M. Wang Q. (2018). Best lighting for jeans, part 1: Optimising colour preference and colour discrimination with multiple correlated colour temperatures. *Light. Res. Technol.* 51 1208–1223. 10.1177/1477153518816125

[B15] Huang Z. Liu Q. Luo M. R. Pointer M. R. Liu A. (2019a). The whiteness of lighting and colour preference, Part 2: A meta-analysis of psychophysical data. *Light. Res. Technol.* 52 23–35. 10.1177/1477153519837946

[B16] Huang Z. Liu Q. Pointer M. R. Luo M. R. Liu A. (2019b). White lighting and colour preference, Part 1: Correlation analysis and metrics validation. *Light. Res. Technol.* 52:18. 10.1177/1477153518824789

[B17] Huang Z. Liu Q. Liu Y. Pointer M. R. Liu A. (2020a). Gender difference in colour preference of lighting: A pilot study. *Light Eng.* 28 111–122. 10.33383/2019-100Vol

[B18] Huang Z. Liu Q. Pointer M. Chen W. Liu Y. Wang Y. (2020b). Color quality evaluation of Chinese bronzeware in typical museum lighting. *J. Opt. Soc. Am. A* 37 A170–A180. 10.1364/JOSAA.381498 32400540

[B19] Huang Z. Liu Q. Luo M. R. Pointer M. R. Liu Y. Wang Y. (2021b). Whiteness and preference perception of white light sources: A case study at 5500 K with positive and negative Duv values. *Optik* 240:166845. 10.1016/j.ijleo.2021.166845

[B20] Huang Z. Chen W. Liu Q. Wang Y. Pointer M. R. Liu Y. (2021a). Towards an optimum colour preference metric for white light sources: A comprehensive investigation based on empirical data. *Opt Exp.* 29 6302–6319. 10.1364/OE.413389 33726155

[B21] Huang Z. Liu Q. Westland S. Pointer M. R. Xiao K. (2017). Light dominates colour preference when correlated colour temperature differs. *Light. Res. Technol.* 50 995–1012. 10.1177/1477153517713542

[B22] Hurlbert A. C. Ling Y. (2007). Biological components of sex differences in color preference. *Current Biology* 17 R623–R625. 10.1016/j.cub.2007.06.022 17714645

[B23] Islam M. S. Dangol R. Hyvarinen M. Bhusal P. Puolakka M. Halonen L. (2013). User preferences for LED lighting in terms of light spectrum. *Light. Res. Technol.* 45 641–665. 10.1177/1477153513475913

[B24] Jiang L. Jin P. Lei P. (2015). Color discrimination metric based on cone cell sensitivity. *Opt Exp.* 23 A741–A751. 10.1364/OE.23.00A741 26072896

[B25] Jost-Boissard S. Avouac P. Fontoynont M. (2015). Assessing the colour quality of LED sources: Naturalness, attractiveness, colourfulness and colour difference. *Light. Res. Technol.* 47 769–794. 10.1177/1477153514555882

[B26] Jost-Boissard S. Fontoynont M. Blanc-Gonnet J. (2009). Perceived lighting quality of LED sources for the presentation of fruit and vegetables. *J. Modern Opt.* 56 1420–1432. 10.1080/09500340903056550

[B27] Khanh T. Q. Bodrogi P. Z. Vinh Q. T. Stojanovic D. (2017). Colour preference, naturalness, vividness and colour quality metrics, Part 1: Experiments in a room. *Light. Res. Technol.* 49 697–713. 10.1177/1477153516643359

[B28] Khanh T. Bodrogi P. (2016). Colour preference, naturalness, vividness and colour quality metrics, Part 3: Experiments with makeup products and analysis of the complete warm white dataset. *Light. Res. Technol.* 50 218–236. 10.1177/1477153516669558

[B29] Khanh T. Bodrogi P. Vinh Q. Stojanovic D. (2016). Colour preference, naturalness, vividness and colour quality metrics, Part 2: Experiments in a viewing booth and analysis of the combined dataset. *Light. Res. Technol.* 49 714–726. 10.1177/1477153516643570

[B30] Liu Q. Huang Z. Pointer M. R. Luo M. R. Xiao K. Westland S. (2017a). Evaluating colour preference of lighting with an empty light booth. *Light. Res. Technol.* 50 1249–1256. 10.1177/1477153517727330

[B31] Liu Q. Huang Z. Xiao K. Pointer M. R. Westland S. Luo M. R. (2017b). Gamut volume index: A color preference metric based on meta-analysis and optimized colour samples. *Opt. Exp.* 25:16378. 10.1364/oe.25.016378 28789142

[B32] Liu Q. Huang Z. Wu B. Liu Y. Wang W. (2018). “Evaluating colour quality of lighting: Why meta-analysis is needed,” in *Proceedings of the 15th China international forum on solid state lighting & 2018 international forum on wide bandgap semiconductors*, Shenzhen.

[B33] Liu Y. Liu Q. Huang Z. Pointer M. R. Rao L. Hou Z. (2020). Optimising colour preference and colour discrimination for jeans under 5500 K light sources with different Duv values. *Optik* 208:163916. 10.1016/j.ijleo.2019.163916

[B34] Melgosa M. García P. A. Gómez-Robledo L. Shamey R. Hinks D. Cui G. (2011). Notes on the application of the standardized residual sum of squares index for the assessment of intra- and inter-observer variability in color-difference experiments. *J. Opt. Soc. Am. A Opt. Image Sci. Vis.* 28 949–953. 10.1364/josaa.28.000949 21532709

[B35] Mizokami Y. Kamesaki C. Ito N. Sakaibara S. Yaguchi H. (2012). Effect of spatial structure on colorfulness adaptation for natural images. *J. Opt. Soc. Am. A* 29:A118. 10.1364/josaa.29.00a118 22330368

[B36] Nascimento S. Masuda O. (2014). Best lighting for visual appreciation of artistic paintings—experiments with real paintings and real illumination. *J. Opt. Soc. Am. A* 31:A214. 10.1364/josaa.31.00a214 24695172

[B37] Nickerson D. Jerome C. W. (1965). Color rendering of light sources: CIE method of specification and its application. *Illum. Eng*. 60, 262–271.

[B38] Ohno Y. Fein M. (2014). “VISION experiment on acceptable and preferred white light chromaticity for lighting,” in *Proceedings of the CIE 2014 lighting quality and energy efficiency*, Kuala Lumpur.

[B39] Ohno Y. Oh S. (2016). “Vision experiment ii on white light chromaticity for lighting,” in *Proceedings of the CIE lighting quality and energy efficiency*, Melbourne.

[B40] Palmer S. E. Schloss K. B. Sammartino J. (2013). Visual aesthetics and human preference. *Annu. Rev. Psychol.* 64 77–107. 10.1146/annurev-psych-120710-100504 23020642

[B41] Panorgias A. Parry N. Mckeefry D. J. Kulikowski J. J. Murray I. J. (2010). Gender differences in peripheral colour vision; a colour-matching study. *Investig. Ophthalmol. Visual Sci.* 51:6288.

[B42] Párraga C. Troscianko T. Tolhurst D. J. (2000). The human visual system is optimised for processing the spatial information in natural visual images. *Curr. Biol.* 10 35–38. 10.1016/s0960-9822(99)00262-6 10660301

[B43] Pearce B. Crichton S. Mackiewicz M. Finlayson G. D. Hurlbert A. (2014). Chromatic illumination discrimination ability reveals that human colour constancy is optimised for blue daylight illuminations. *PLoS One* 9:e87989. 10.1371/journal.pone.0087989 24586299 PMC3929610

[B44] Pirenne M. H. (1948). Vision and the eye. *J. Sci.* 7:222. 10.1111/j.1751-1097.1968.tb07413.x

[B45] Royer M. P. Wilkerson A. M. Wei M. Houser K. W. Davis R. G. (2016). Human perceptions of colour rendition vary with average fidelity, average gamut, and gamut shape. *Light. Res. Technol.* 49 966–991. 10.1177/1477153516663615

[B46] Schwarzkopf D. S. Song C. Rees G. (2011). The surface area of human V1 predicts the subjective experience of object size. *Nat. Neurosci.* 14 28–30. 10.1038/nn.2706 21131954 PMC3012031

[B47] Smet K. A. G. (2018). Two neutral white illumination loci based on unique white rating and degree of chromatic adaptation. *Leukos* 14 55–67. 10.1080/15502724.2017.1385400

[B48] Smet K. A. G. Ryckaert W. R. Pointer M. R. Deconinck G. Hanselaer P. (2010). Memory colours and colour quality evaluation of conventional and solid-state lamps. *Opt. Exp.* 18:26229. 10.1364/oe.18.026229 21164972

[B49] Smet K. Ryckaert W. R. Pointer M. R. Deconinck G. Hanselaer P. (2011). Correlation between color quality metric predictions and visual appreciation of light sources. *Opt. Exp.* 19 8151–8166. 10.1364/oe.19.008151 21643065

[B50] Szabó F. Bodrogi P. Schanda J. (2009). A colour harmony rendering index based on predictions of colour harmony impression. *Light. Res. Technol.* 41 165–182. 10.1177/1477153509103067

[B51] Tan K. W. Stephen I. D. (2019). Skin color preferences in a Malaysian Chinese population. *Front. Psychol.* 10:1352. 10.3389/fpsyg.2019.01352 31275195 PMC6594203

[B52] Thornton W. A. (1972). Color-discrimination index. *J. Opt. Soc. Am.* 62 191–194. 10.1364/JOSA.62.000191 5009385

[B53] Thornton W. A. (1974). A validation of the color-preference index. *J. Illum. Eng. Soc.* 4 48–52. 10.1080/00994480.1974.10732288

[B54] Vanston J. E. Strother L. (2016). Sex differences in the human visual system. *J. Neurosci. Res.* 95 617–625. 10.1002/jnr.23895 27870438

[B55] Wang Y. Wei M. (2018). Preference among light sources with different D_(uv) but similar colour rendition: A pilot study. *Light. Res. Technol.* 50 1013–1023. 10.1177/1477153517712552

[B56] Wang Y. Liu Q. Gao W. Pointer M. R. Huang Z. Chen W. (2020). Interactive effect of illuminance and correlated colour temperature on colour preference and degree of white light sensation for Chinese observers. *Optik* 224:165675. 10.1016/j.ijleo.2020.165675

[B57] Wei M. Houser K. W. (2015). What is the cause of apparent preference for sources with chromaticity below the blackbody locus? *Leukos* 12 95–99. 10.1080/15502724.2015.1029131

[B58] Wei M. Houser K. W. Allen G. R. Beers W. W. J. L. (2014). Color preference under LEDs with diminished yellow emission. *Leukos* 10 119–131. 10.1080/15502724.2013.865212

[B59] Wei M. Houser K. W. David A. D. J. Krames M. R. (2016). Colour gamut size and shape influence colour preference. *Light. Res. Technol.* 49 992–1014. 10.1177/1477153516651472

[B60] Zhai Q. Y. Luo M. R. Liu X. Y. (2014). The impact of illuminance and colour temperature on viewing fine art paintings under LED lighting. *Light. Res. Technol.* 47 795–809. 10.1177/1477153514541832

